# The link between glycemic control measures and eye microvascular complications in a clinical cohort of type 2 diabetes with microRNA-223-3p signature

**DOI:** 10.1186/s12967-023-03893-2

**Published:** 2023-03-03

**Authors:** Sahar I. Da’as, Ikhlak Ahmed, Waseem H. Hasan, Doua A. Abdelrahman, Elbay Aliyev, Sabah Nisar, Ajaz Ahmad Bhat, Mugdha V. Joglekar, Anandwardhan A. Hardikar, Khalid A. Fakhro, Ammira S. Al-Shabeeb Akil

**Affiliations:** 1 Department of Human Genetics-Precision Medicine in Diabetes, Obesity and Cancer Program, Sidra Medicine, P.O. Box 26999, Doha, Qatar; 2 Laboratory of Genomic Medicine-Precision Medicine Program, Sidra Medicine, P.O. Box 26999, Doha, Qatar; 3 Zebrafish Functional Genomics, Integrated Genomic Services Core Facility, Research Branch, Sidra Medicine, P.O. Box 26999, Doha, Qatar; 4 College of Health and Life Sciences, Hamad Bin Khalifa University, P.O. Box 34110, Doha, Qatar; 5 Diabetes and Islet Biology Group, School of Medicine, Western Sydney University, Narellan Road & Gilchrist Drive, Campbelltown, NSW 2560 Australia; 6 Department of Science and Environment, Roskilde University, Universitetsvej 1, 4000 Roskilde, Denmark; 7 Department of Genetic Medicine, Weill Cornell Medical College, P.O. Box 24144, Doha, Qatar

**Keywords:** Type 2 diabetes, Diabetic retinopathy, miR-223-3p, Zebrafish modeling, Hyperglycemia, Functional validation

## Abstract

**Background:**

Type 2 diabetes (T2D) is a critical healthcare challenge and priority in Qatar which is listed amongst the top 10 countries in the world, with its prevalence presently at 17% double the global average. MicroRNAs (miRNAs) are implicated in the pathogenesis of (T2D) and long-term microvascular complications including diabetic retinopathy (DR).

**Methods:**

In this study, a T2D cohort that accurately matches the characteristics of the general population was employed to find microRNA (miRNA) signatures that are correlated with glycemic and β cell function measurements. Targeted miRNA profiling was performed in (471) T2D individuals with or without DR and (491) (non-diabetic) healthy controls from the Qatar Biobank. Discovery analysis identified 20 differentially expressed miRNAs in T2D compared to controls, of which miR-223-3p was significantly upregulated (fold change:5.16, p = 3.6e−02) and positively correlated with glucose and hemoglobin A1c (HbA1c) levels (p-value = 9.88e−04 and 1.64e−05, respectively), but did not show any significant associations with insulin or C-peptide. Accordingly, we performed functional validation using a miR-223-3p mimic (overexpression) under control and hyperglycemia-induced conditions in a zebrafish model.

**Results:**

Over-expression of miR-223-3p alone was associated with significantly higher glucose (42.7 mg/dL, n = 75 vs 38.7 mg/dL, n = 75, p = 0.02) and degenerated retinal vasculature, and altered retinal morphology involving changes in the ganglion cell layer and inner and outer nuclear layers. Assessment of retinal angiogenesis revealed significant upregulation in the expression of vascular endothelial growth factor and its receptors, including kinase insert domain receptor. Further, the pancreatic markers, pancreatic and duodenal homeobox 1, and the insulin gene expressions were upregulated in the miR-223-3p group.

**Conclusion:**

Our zebrafish model validates a novel correlation between miR-223-3p and DR development. Targeting miR-223-3p in T2D patients may serve as a promising therapeutic strategy to control DR in at-risk individuals.

**Supplementary Information:**

The online version contains supplementary material available at 10.1186/s12967-023-03893-2.

## Introduction

Type 2 diabetes is characterized by metabolic dysregulation, particularly abnormal glucose metabolism, accompanied by various long-term complications [1]. The associated complications include diabetic nephropathy, neuropathy, and retinopathy (DR). Despite current treatments, the global increase in diabetes and related complications, including vascular and neurological conditions, is alarming. Over 415 million people worldwide have diabetes, including 46% lacking the diagnosis (based on International Diabetes Federation 2019 data) [2]. In Qatar, an alarming increase of 130% is predicted by 2030 [3]. Genetic factors, a sedentary lifestyle, and an unhealthy diet are major contributors to the global diabetes epidemic. Type 2 diabetes causes significant morbidity and mortality due to various micro-and macrovascular complications [4].

Among the microvascular complications in type 2 diabetes, DR has one of the highest incidences. DR is a sight-threatening neurodegenerative microvascular retinal complication of both T1D and T2D causing a substantial healthcare burden [5, 6]. In 2019, the global prevalence of DR was 27%, and it is expected to double by 2025 [7, 8]. DR results from abnormal vascularization of retinal blood vessels and can be classified into non-proliferative and proliferative DR (NPDR and PDR). In DR, exposure to high glucose levels increases the vascular permeability of the blood-retinal barrier, causing the leakage of fluids and lipids into the retina. The precise molecular mechanism underlying the pathophysiology of DR is still unclear; therefore, understanding the molecular and physiological changes induced by high glucose exposure is crucial.

The prognostic value of many metabolic biomarkers, including glycosylated hemoglobinA1c (HbA1c), glucose, high-density lipoprotein (HDL), and triglycerides, is limited [9]. Current clinical interventions, including intravitreal steroids, vascular endothelial growth factor (VEGF) injections, and laser and blood glucose control, have been unresponsive in most patients. This emphasizes the need for regulatory biomarkers at the molecular level to target inflammatory responses at very early stages in diabetic patients. Identifying highly sensitive and specific biomarkers associated with T2D and related complications is urgent. We aim to profile biomarkers as tools for personalized treatment of T2D-related complications.

miRNAs are significant in maintaining metabolic homeostasis and regulating autocrine and endocrine gene expression [10]. Their role as biomarkers in T2D and correlated complications is reported e.g., altered miRNA expression causes DR-related micro-vascularization and pathogenesis [11]. Profiling the miRNAs linked to T2D with or without DR and investigating the related pathways and their effect on the disease is critical. But the miRNA association profiles vary in populations owing to genetic and environmental differences. The scarcity of extensive research on circulating biomarkers for DR, particularly in the Middle Eastern and North African populations (MENA), has led us to construct miRNA profiles associated with T2D [2, 12]. Additionally, the zebrafish is a well-established animal model organism to study vascular development and function processes in various diseases, including metabolic disorders, hyperglycemia-generated conditions, and diabetes-induced complications [13, 14].

Classical T2D is triggered by insulin resistance and β-cell dysfunction resulting from β-cell loss. Despite the availability of traditional therapies for T2D, the prevalence of complications such as diabetic retinopathy, nephropathy, or neuropathy [15, 16] is still increasing in all age groups [15]. Multiple animal models have proven to be great tools for novel discoveries to improve the efficiency of existing T2D therapies and identify unknown disease mechanisms. Zebrafish are the go-to model for observing organ development, morphology, and function in normal and hyperglycemia conditions. It allows for high throughput screenings through in vivo imaging.

Zebrafish and humans share 70% of their protein-coding genes, and 84% of the human disease-causing genes have a zebrafish ortholog [16]. In addition, zebrafish share many of the same tissues and organs as humans. Several genes and vital metabolic processes needed to develop these traits are substantially conserved between humans and zebrafish. We anticipate that mimicking these human disease genes in zebrafish would enable researchers to gather vital biological data on how these genes work and perhaps identify novel therapeutic targets. The fact that miR-223-3p is highly conserved across species and that the mature miR-223-3p sequences of zebrafish and humans are 100% identical is more evidence for the zebrafish and humans sharing common genetic makeup. Due to its transparency, zebrafish has recently become a useful model for studying diabetic consequences, such as diabetic retinopathy and nephropathy [17]. The simplicity of elevating the glucose levels and high number of produced embryos in zebrafish lead to maximizing their suitability for studying these diabetes-correlated complications. It was reported that cultivating the animals in high glucose medium for 3–6 weeks produced hyperglycemia in adult zebrafish [18–20], amenable to revealing pathophysiological mechanisms with other mammals [21, 22]. Recent data indicate that the regulation of glucose metabolism in zebrafish, through the production of insulin, is similar to mammalian models, and many of the genes involved in regulating blood glucose levels have been identified in zebrafish. The genes of zebrafish insulin and glucagon, as well as other important proteins in the regulation of glucose metabolism, have been identified and demonstrate similar regulation patterns and activity as seen in mammalian counterparts [23–25].

In T2D, diet-induced obesity models have been established in zebrafish and were sophisticated enough to reveal pathophysiological mechanisms with other mammals [21, 22]. For example, the role of leptin in zebrafish glucose homeostasis seems to be similar to the events that occur in mammals. Downregulation of Leptin-receptor in oversized zebrafish had resulted in elevated insulin levels after feeding and a diabetes-like lesion in wound healing. However, the leptin hormone does not influence adiposity in the zebrafish model [26]. As well, a skeletal muscle insulin resistance transgenic mice model has been established with the exhibition of impaired glucose clearance and tolerance. Maddison et al. established a transgenic zebrafish line with skeletal muscle insulin resistance. After overfeeding, the transgenic fish display impaired glucose clearance and intolerance [27, 28].

In this cohort, several miRNAs, including miR-223-3p, are reported to be associated with DR [29]. MiR-223-3p has been implicated in several diseases, such as diabetes and its complications (e.g., DR). It is crucial in maintaining functional β-cell mass by regulating forkhead box O1 (*FOXO1*) and SRY-box 6 (*SOX6*) signaling [30]. Its expression is also correlated with high-glucose-induced cell proliferation [31]. The development and function of organs involved in insulin metabolism and homeostasis, e.g. pancreas, are conserved in zebrafish, making it a valuable model for investigating metabolic pathologies [32]. In the present study, functional validation in zebrafish confirmed increased expression and association of miR-223-3p in diabetes and DR, suggesting its role as a biomarker and potential therapeutic target for metabolic disorders. We designed the present study to envisage the expression profiles of 51 pre-selected (based on previous experimental results) circulating miRNA targets with respect to our phenotypic classification, i.e., diabetic, NPDR, and PDR individuals in the Qatari population. Our study revealed the role of miR-223-3p in the early stages of retinopathy and its relationship to hyperglycemic conditions within a unique zebrafish model.

## Materials and methods

### Study population, available data and clinical samples

The study comprises 962 individuals, 471 with type 2 diabetes and 491 healthy non-diabetic controls, enrolled through the Qatar Biobank (QBB) and the Qatar Genome Program (QGP). All participants in the study signed informed consent. Upon enrollment, clinical and laboratory evaluations were performed (Additional file 1). Anthropometric measurements and plasma samples were obtained from the QBB to perform the initial analysis and generate data for validation studies. The retinal images of all study subjects were acquired using a Topcon TRC-NW6S retinal camera. A qualified ophthalmologist selected the best quality images (2) from each individual and classified as normal, NPDR, or PDR.

### Circulating RNA extraction

RNA was extracted from plasma using the PureLink RNA mini kit (Thermo Fisher Scientific, CA, US) following the manufacturer’s protocol. RNA concentration was measured using a nanodrop spectrophotometer (Invitrogen), and the samples were stored at − 80 °C until further use.

### cDNA synthesis and pre-amplification

cDNA synthesis and pre-amplification for reverse transcription (RT)-PCR analysis was performed using a low sample input protocol, following the manufacturer’s protocol (Superscript IV First-Strand Synthesis System, Cat# 18091050, Invitrogen).

### Open array miRNA profiling

A TaqMan® custom OpenArray™ panel included 51 potential biomarkers reported by our group in discovery analyses related to islet/vascular health [33–35]. A QuantStudio12K Flex® system with an Accufill® robotic platform using the low sample input protocol as described by our group earlier [36, 37].

### miRNAs gene expression analysis

Real-time PCR was used to assay all 51 plasma-enriched microRNAs. The data were analyzed for differential expression using custom scripts and the PCR package in R [38]. Normalization of quantification cycles (Ct) was performed using a spike-in exogenous control, ath-miR-159a, to calibrate the Ct values for any unintentional variation in sample input, as described earlier [33, 39]. Expression was calibrated using the spike-in control (ath-miR159a) and fold changes were computed against the mean expression of normal subjects based on the 2^(−∆∆ct)^ [40] method.

### Association analysis

We tested the associations of two clinical phenotypes (HbA1c and C-peptide) with miRNA expression. To ensure normality of the outcomes, the continuous variables HbA1c and C-peptide were rank-based inverse normal transformed. Multivariate generalized additive models (GAMs) were employed to model the association between HbA1c, C-peptide, and miRNA expression. We used generalized additive models to relax the linearity assumption while maintaining maximum interpretability. GAMs allowed us to fit a non-linear function to each variable, allowing automated modelling of non-linear relationships. All analyses were adjusted for age, gender, and body mass index (BMI).

We used a chi-square test to identify gender associations with the disease. To test the age-based association, we also constructed age groups using the quartiles of the continuous parameter age. We used a chi-square test for associations between diabetic/non-diabetic status with the constructed age groups. Any association with a p-value less than 0.05 was considered significant. All statistical analyses were performed using the statistical software R version 3.6.1.

### MiRNA functional validation experiments

#### MiRNA selection for functional validation in a zebrafish model

Expression analysis of the selected miRNA revealed differential expression of many miRNAs in control and diabetic individuals with and without DR, including miR-223-3p, which was upregulated in diabetic patients. We used the miRbase database to identify the zebrafish ortholog of human miR-223-3p (miRBase ID MIMAT0001290).

#### Zebrafish maintenance and care

Zebrafish (*Danio rerio*) husbandry was performed following standard protocols [41] and maintained as approved by the local Animal Care Committee (Qatar Foundation–EVMC-2020-006). Zebrafish reporter line, Tg (fli1a:GFP:mitfa,roy) with vasculature expressing green fluorescent protein (GFP), was used to visualize the developing vasculature.

#### MiRNA-223 functional validation in zebrafish model

To investigate the upregulation of miR-223-3p, a zebrafish miR-223-3p mimic (Thermo Fisher Cat# 4464066) was dissolved in RNAse-free water to a stock concentration of 50 μM. Adult male and female Zebrafish were set up for breeding. The Zebrafish embryos were collected. Using a microcapillary filament needle, the miR-223-3p mimic reagent was loaded into the needle with a fine tip, and then Zebrafish embryos were injected with 2 nL of 1.5 μM miR-223-3p mimic reagent at a one-to four-cell stage using a picoliter injector (Harvard Apparatus). The injection procedure was performed as previously described [42].

#### Induction of hyperglycemia in zebrafish

The control- and miR-223-3p mimic-injected zebrafish groups were exposed to 0%, 2%, and 5% D-glucose (Cat# G7021) in egg media from 3-h post-fertilization (hpf) until 3-days post fertilization (dpf) following a modified methodology. Zebrafish embryos of the transgenic reporter line (fli1a:GFP::mitfa,roy) with vasculature expressing GFP were placed in 30 mL of egg media in Petri dishes with various concentrations of d-glucose (0%, 2%, or 5% w/v) and maintained at 28.5 °C. The incubation media was changed every 24 h until day 3. For total glucose measurement, embryos (n = 20) were transferred to microfuge vials, immersed in ice, and rinsed three times in egg media. The media was completely aspirated, embryos homogenized using a hand homogenizer, and centrifuged at 14,000×*g* for 2 min. A supernatant volume of 1.5 μL was used to measure total free-glucose levels using a glucometer (Accu-Chek Performa Nano).

#### Analysis of retinal vasculature

We examined changes in the retinal blood vessels of the miR-223-3p injected embryos under the three different glucose conditions by comparing them to the control groups (imaged at 3 dpf). Representative images were used to evaluate miR-223-3p effect on the eye: the diameter of the eye was traced, and the defined area was analyzed using DanioScope software. Eyes were examined for impaired vasculature development and classified according to three phenotypes: normal, mild, and severe. Three independent evaluations were performed in a blinded manner in each larval group (n = 10).

#### Zebrafish embryo retinal histology

Embryos in different groups (raised in pre-specified glucose conditions and controls) were fixed and sectioned to examine the effect of the miR-223-3p mimic on the development of cellular layers of the eye. Hematoxylin and eosin staining was performed according to a standard protocol [43].

#### Real-time quantitative PCR for miRNA-223 validation

Pooled whole zebrafish larvae (50 larvae) at 3dpf of each experimental group were used for total RNA extraction (Qiagen, Cat #). The extracted total RNA was used to perform quantitative RT-PCR using TaqMan microRNA assay stem-loop primers for mature miR-223-3p (Thermo Fisher Scientific, Assay ID# 000526) and TaqMan Universal PCR Master Mix (Thermo Fisher Scientific) following the manufacturer’s protocol.

#### Real-time quantitative PCR for vasculature and apoptosis genes

cDNA synthesis was done using SuperScript IV Reverse Transcription kit (Thermo Fisher Scientific, Cat #18091050) following the manufacturer’s protocol. The qPCR reactions were performed using Power SYBR Green Master Mix (Thermo Fisher Scientific, Cat#A25742) on the Quantstudio 12K real-time PCR platform (Applied Biosystems) to detect angiogenesis (vasculature) markers, *VEGF* and the two structurally related receptors, *FLT-1* (VEGF receptor-1) and *KDR* (VEGF receptor-2); pancreatic markers insulin (*INS*), pancreatic and duodenal homeobox 1 (*PDX1*), and apoptosis markers CASPASE 8 (*CASP8*) and B-cell lymphoma 2 *BCL2* (Additional file 1: Table S2), and the expression of the markers were analyzed using the ddCT method. The data were normalized using the housekeeping gene, elongation factor 1-alpha 1 (*EF1A*).

#### Statistical analysis

Each result is presented as the mean of at least three experiments. Multiple comparisons were analyzed with one-way ANOVA using GraphPad Prism (version 8.4.3). The level of significant difference between groups was expressed using p-values, and a p-value of < 0.05 represented statistical significance. Differential gene expression between patient and control groups was analyzed using the Kruskal–Wallis test for multiple comparisons of the mean and Wilcoxon rank-sum test for each pair of comparisons. Chi-square test was used to analyse age and gender-based association with miR-223-3p expression and has been described in “Association analysis” section.

## Results

### Anthropometric and clinical characteristics of the Qatar Biobank cohort

The cohort of type 2 diabetic patients and their matched controls were selected from the QBB [44] based on their clinical characteristics and anthropometric measurements. All clinical and anthropometric measurements relevant to type 2 diabetes including HbA1c, C-peptide, insulin, low-density lipoprotein, high-density lipoprotein, total cholesterol, triglycerides, and BMI, are summarized for mean values by gender to identify any outliers in the data (Additional file 1: Table S1). These clinical traits were closely matched between males and females for both patient and control cohorts, indicating the absence of any outlier groups in our dataset. HbA1c, C-peptide, insulin, and BMI, type 2 diabetes-associated parameters, were substantially high in male and female diabetic patients compared to the control subjects.

### miRNA expression profiling and association with clinical parameters

**Fig. 1:**
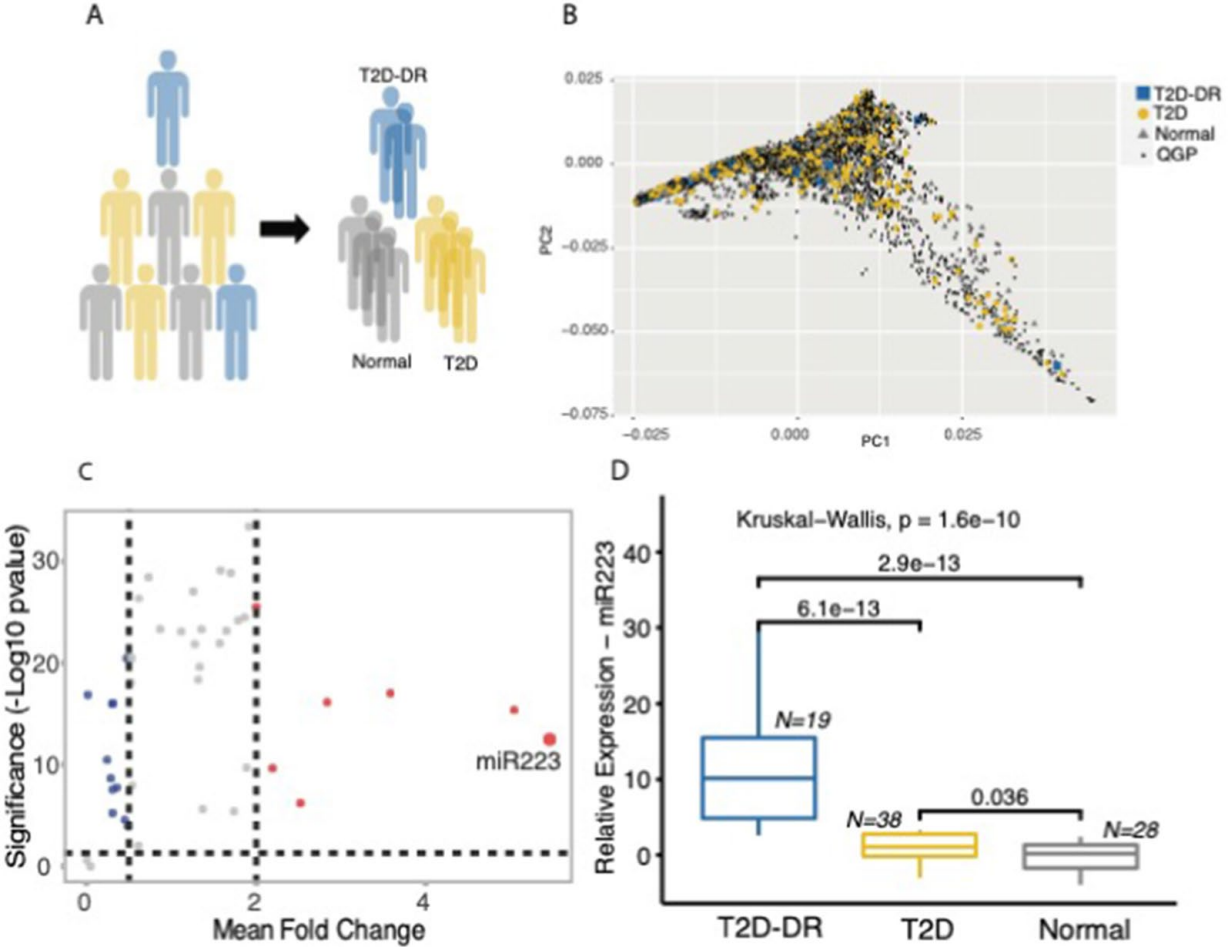
miR-223-3p is upregulated in individuals with type 2 diabetes and diabetic retinopathy. **A** A sub-cohort of individuals selected from the Qatar Genome Programme (QGP) participants were stratified into T2D patients and Normal controls based on self-reporting of a physician diagnosis for diabetes or HbA1c levels greater than 6.5%. T2D individuals with diabetic retinopathy (T2D-DR) were identified by examining the retinal images. **B** Principal component analysis based on biallelic germline variants presents the distribution of case- and control-study samples across the nationwide QGP population with no selection bias in the representative sample. **C** mi-R223 was identified as the top-upregulated miRNA in our target panel with elevated expression in T2D. **D** Amongst T2D individuals, miR-223-3p expression was significantly higher in plasma from individuals with DR (T2D-DR) compared to Controls and T2D individuals without DR. Boxplots show the distribution of relative expression for miR-223-3p across all study samples

We selected a sub-cohort of 962 human subjects from a larger cohort of ~ 6000 individuals that have been whole-genome sequenced in the framework of the Qatar Genome Programme. These subjects were stratified into T2D or normal controls based on self-reported physician diagnosis of T2D or HbA1c levels (Fig. 1). A qualified ophthalmologist further examined their retinal images at Sidra Medicine ophthalmology clinic to identify cases who have progressed to diabetic retinopathy (T2D-DR). To assess whether participants in our representative sample would represent the characteristics of the entire population, we computed principal components based on biallelic, autosomal markers for the entire cohort of ~ 6000 subjects. The PCA plot in Fig. 1B shows that our sub-cohort of 962 subjects represents the target population as cases and controls do not form specific clusters but are spread throughout and indistinguishable from non-participants. The expression of 51 plasma-enriched miRNAs from T2D and T2D-DR patient cohorts was compared to the control group and with each other to investigate the differential expression patterns of the selected miRNAs. Samples that passed the quality filters and showed the expression of the assayed miRNA were used in the calculations. We identified 20 circulating miRNAs showing altered expression at least twofold between type 2 diabetics and healthy controls, 7 miRNAs being upregulated and 13 downregulated in patients. Among the differentially regulated miRNAs, miR-223-3p was the most upregulated showing significant differences in mean expression (Kruskal–Wallis test; p = 1.6e−10) among patients with retinopathic type 2 diabetes, non-retinopathic type 2 diabetes, and control subjects. As shown in (Fig. 1), significant upregulation was detected for miR-223-3p in DR patients compared to non-DR type 2 diabetes patients (Wilcoxon rank-sum test; p = 6.1e−13) and control subjects (Wilcoxon rank sum test; p = 2.9e−13). The highly elevated levels of hsa-miR-223-3p in DR patients suggest miR-223-3p as a potential indicator of the development of retinopathy and a useful plasma biomarker for disease prognosis in type 2 diabetes patients.

**Fig. 2:**
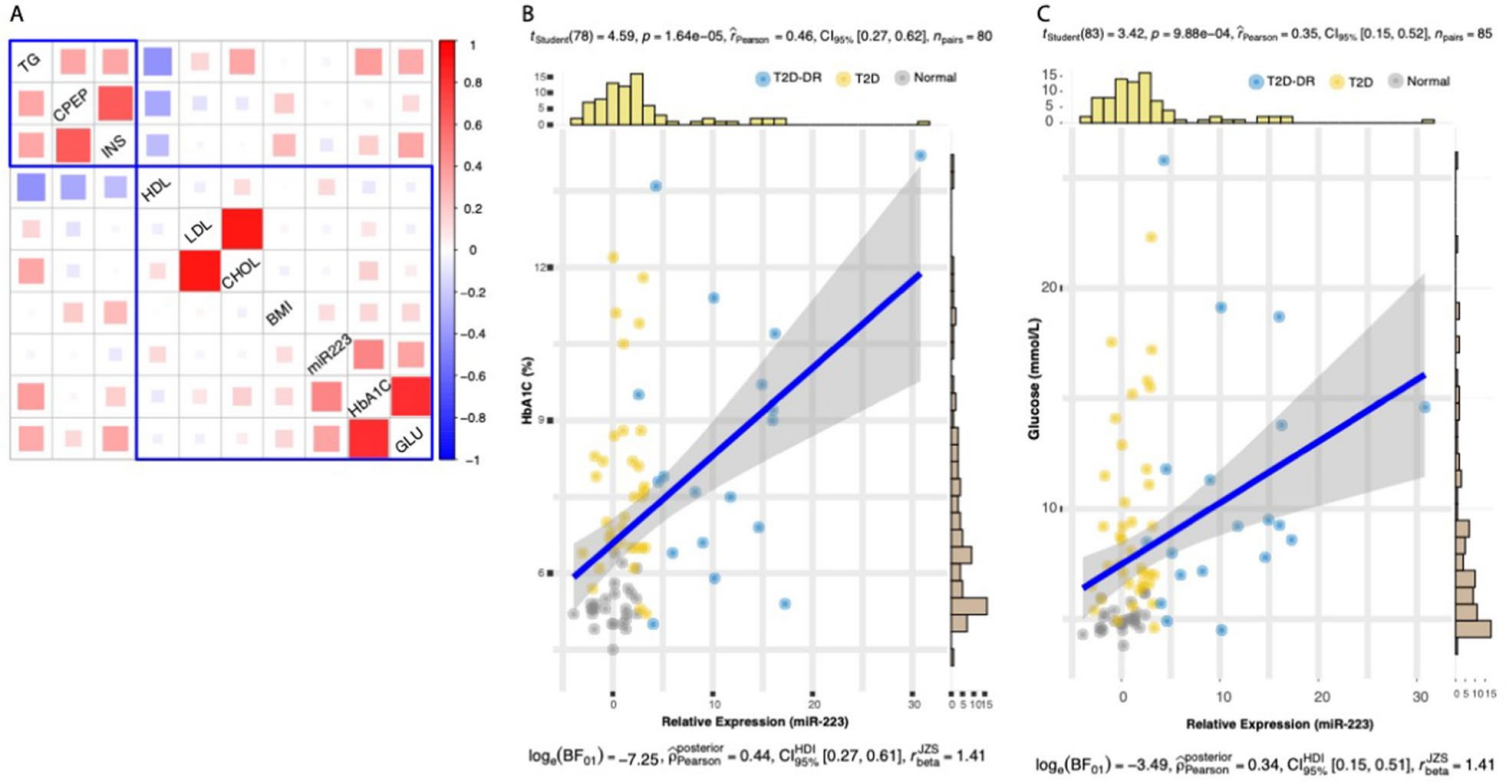
Association of miR-223-3p expression with clinical characteristics of diabetes. **A** Heatmap of the hierarchically clustered Pearson correlation coefficients. The rectangles demarcate two major clusters of trait associations as identified through hierarchical clustering method. Mi-R223 expression was observed to significantly correlate with **B** plasma HbA1C and **C** glucose levels. The statistics for the Pearson’s correlation test are shown at the top of each scatterplot, and the Bayes Factor analysis is shown at the bottom. The scatterplots and their associated statics were calculated through ggstatsplot package [45] in R. *TG* triglycerides, *CPEP* C-peptide, *INS* insulin, *HDL* high-density lipoprotein, *LDL* low-density lipoprotein, *CHOL* total cholesterol, *BMI* body mass index, *miR-223-3p* microRNA miR-223-3p, *HbA1c* hemoglobin A1c, *GLU* glucose

Analysis of the association of 51 miRNA expression with clinical parameters, HbA1c and C-peptide, using generalized additive linear models (adjusted for age, gender, and BMI) revealed significant association of six miRNAs with HbA1c (p > 0.05) and two with C-peptide in type 2 diabetic patients. Interestingly, miR-223-3p showed a significant association with HbA1c (p = 4e−02) but not with C-peptide (Table 1). Through correlation analysis, we further investigated the relationship between miR-223-3p expression and nine other clinical characteristics of T2D in our cohort (Fig. 2). The heatmap of the correlation coefficients shows two major clusters for these trait associations—one formed by triglycerides, C-peptide, and insulin while as the other comprised of miR-223-3p with HDL, LDL, Cholesterol, BMI, HbA1C and glucose. Out of the nine traits, miR-223-3p showed a positive correlation with five of them, however the association was statistically significant only for plasma HbA1C and glucose levels (r_Pearson_ = 0.46 and 0.35, respectively; p-value = 1.64e−05 and 9.88e−04).

**Table 1:** Associations between clinical phenotypes HbA1c and C-peptide and miRNA expression. miR223 shows a significant association with HbA1c levels

miRNA	p-value (HbA1c)	p-value (C-peptide)
miR-27a	3.3E−03^a^	0.51
miR-92a	4.1E−03^a^	0.50
miR-376c	8.6E−03^a^	0.54
miR-451	2.0E−02^a^	0.95
miR-1	4.1E−02^a^	0.86
miR-223	4.5E−02^a^	1
miR-24	0.48	6.8E−06^a^
miR-1274A	0.72	6.6E−03^a^

^a^Significant association

### miRNA functional validation in a zebrafish model

#### Conservation of miR-223-3p

In our Qatari cohort study, we identified miR-223-3p as the most significantly upregulated miRNA in participants with type 2 diabetes (Fig. 1). To study the impact of elevated miR-223-3p levels on retinopathy in an in vivo model, we used the miRbase database to identify the zebrafish ortholog (miRBase ID MIMAT0001290). Our alignment showed miR-223-3p to be highly conserved among species, with 100% identity between the zebrafish and the human mature miR-223-3p sequences (Additional file 1: Fig. S1).

#### Functional validation of miR-223-3p in a zebrafish model

**Fig. 3:**
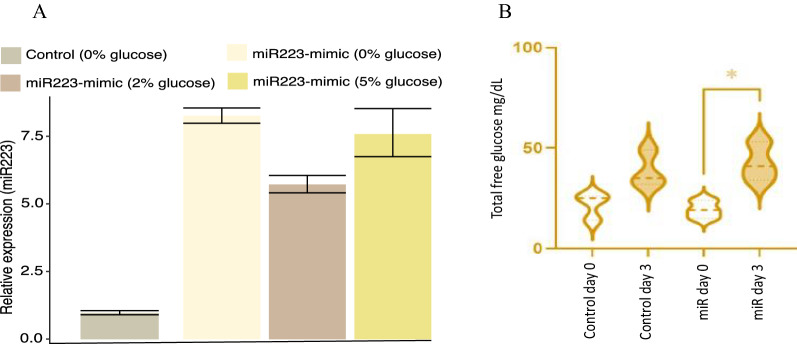
Functional validation of miR-223-3p in a hyperglycemic zebrafish model. **A** Fold change increase in miR-223-3p mimic in various glucose concentrations compared to control group at 0% glucose. **B** Overexpression of miR-223-3p resulted in a significant increase in total glucose concentrations in zebrafish. The expression of miR-223-3p resulted in a significant increase in total glucose levels when measured at 3 days old (day 3) compared to the baseline glucose levels at 3 h post-fertilization (3hpf, day 0), n = 75 larvae per group. Data represent n = 3 separate experiments and are presented with a mean (dashed lines) and quartiles (dotted lines) for the distribution observed. Total glucose levels were measured using Accu-Check Performa Nano. Statistical analysis was conducted using t-test Graph Pad version 9.0

The miR-223-3p mimic injections into 1–4 cell stage embryos resulted in six- to eightfold miR-223-3p overexpression within the zebrafish larvae (Fig. 3A). It’s interesting to note that the miR-223-3p levels were unaffected by the different glucose incubation settings and that they rose by ~ eightfold in both the 0% and 5% incubated groups in comparison to the control group baseline. However, when the control zebrafish were incubated in glucose conditions, the expression of miR-223-3p was decreased (Additional file 1: Fig. S2). Injection of miR-223-3p mimic had no effect on zebrafish survival at varying glucose concentrations—0%, 2% or 5%, compared to controls under matching glucose conditions (Additional file 1: Fig. S2A). Nevertheless, induction of hyperglycemia at 2 and 5% glucose negatively regulated zebrafish survival in both the control and mimic miR-223-3p group in a dose-dependent manner.

**Fig. 4:**
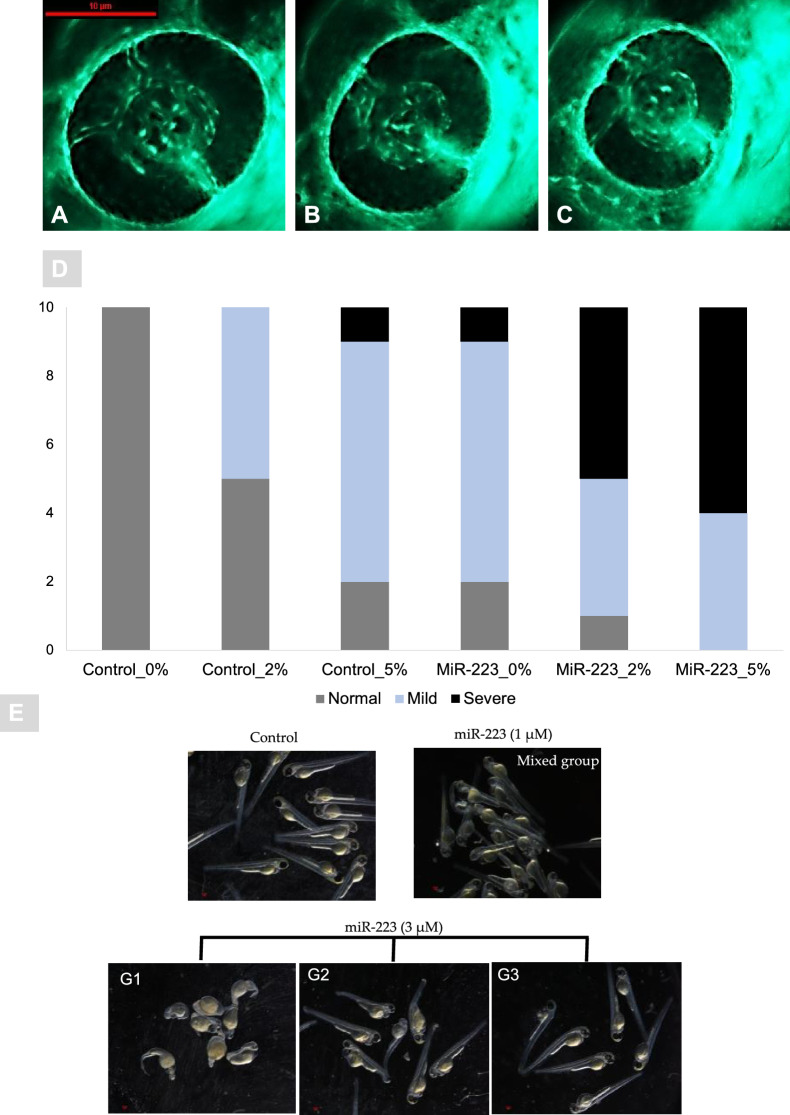
miR-223-3p resulted in aberrant eye vasculature sprout formation. Normal zebrafish eye vasculature development is characterized by three clearly branched blood vessels (indicated by white arrowheads). Exposure to miR-223-3p mimic restricted the growth of these three vessels. The examined groups were scored as three subclasses: normal, mildly affected, and severely affected. **A**–**C** Representative images of Tg (fli1a:roy,mitf:GFP) with vasculature expressing green fluorescent protein; **A** normal, **B** mild, **C** severe. The blood vessels displayed abnormal sprouting in mildly and severely affected eyes. Both miR-223-3p (MiR0%G, MiR 2%G, MiR 5%G) and hyperglycemia conditions (control 2%G, control 5%G) resulted in a significant alteration to the developing of blood vessels compared to control with no glucose incubation (control 0%G). Images were obtained using Lumar 12 stereomicroscope (Zeiss Microscopy) and a Nikon camera at ×100 magnification; scale bar, 10 µm. A total number of larvae measured was n = 10 per group. Statistical analysis was conducted using chi-square. **D** Bar plots showing the distribution of normal, mild, and severe phenotypes in control and miR-223-3p groups in different hyperglycemic conditions (0% G, 2% G, 5% G). **E** Representative images of injected miR-223-3p embryos into the one-cell stage, with different dose titration levels (1, and 3 μM) in the absence of glucose. The embryos at three days post fertilization were presented with severe developmental defects with 3 μM titration and were classified into 3 groups (G1, G2, and G3) depending on the severity and percentages of abnormally developed larvae. Compared to the control group, the larvae in 1 μM appeared with mixed effects that were less prominent than 3 μM groups. Representative Images were captured using Lumar V.12 stereomicroscope and Nikon camera at ×25

Development of zebrafish maintained at different glucose concentrations resulted in a negative effect in the case of miR-223-3p injection compared to controls in matching glucose conditions (Additional file 1: Fig. S2B and Fig. 4E). Zebrafish development was scored in three phenotype categories (G1; severe impairment, G2; moderate impairment, and G3; normal). The percentages of normally developed larvae (G3) in the miR-223-3p mimic group were 68.6%, 40.8%, and 38.6%, while those in the control group were 100%, 56%, and 76% at 0%, 2%, and 5% glucose conditions, respectively (p = 0.05). A significant change in development was observed in the miR-223-3p-mimic-injected group maintained in 2% and 5% glucose (p < 0.0001; Additional file 1: Fig. S2). Further examinations and assessment of the miR-223-3p effect on the glucose levels, eye development, and molecular markers were performed only using G3-classified larvae (similar to the gross morphology of the control group).

#### Increased miR-223-3p levels associated with elevated total free-glucose throughout zebrafish development

To reveal the impact of significantly elevated miR-223-3p levels on glucose metabolism, we measured the glucose levels within the zebrafish model throughout its development. The total free glucose levels were considerably increased as a result of miR-223-3p mimic injections (Group MiR-223-3p_0%) in the 0% glucose condition (p = 0.02) (Fig. 3B). The total free-glucose levels in 3-day-old zebrafish measured 42.7 mg/dL (average total glucose on day 3) compared with 19.3 mg/dL glucose baseline in zebrafish at 3 hpf (Group MiR-223-3p_0%, on day 0) (Fig. 3B). The zebrafish groups were examined for whole-lysate total free-glucose, and the miR-223-3p mimic group showed 42.7, 56.3, and 84.7 mg/dL on day 3 of development for the 0%, 2%, and 5% glucose conditions, respectively, compared to the control groups in the matched glucose condition, which showed 38.7, 52.3, and 82 mg/dL (Additional file 1: Fig. S3). These results confirmed the association between increased miR-223-3p levels and increased glucose levels during development.

#### High miR-223-3p levels induced ocular defects

**Fig. 5:**
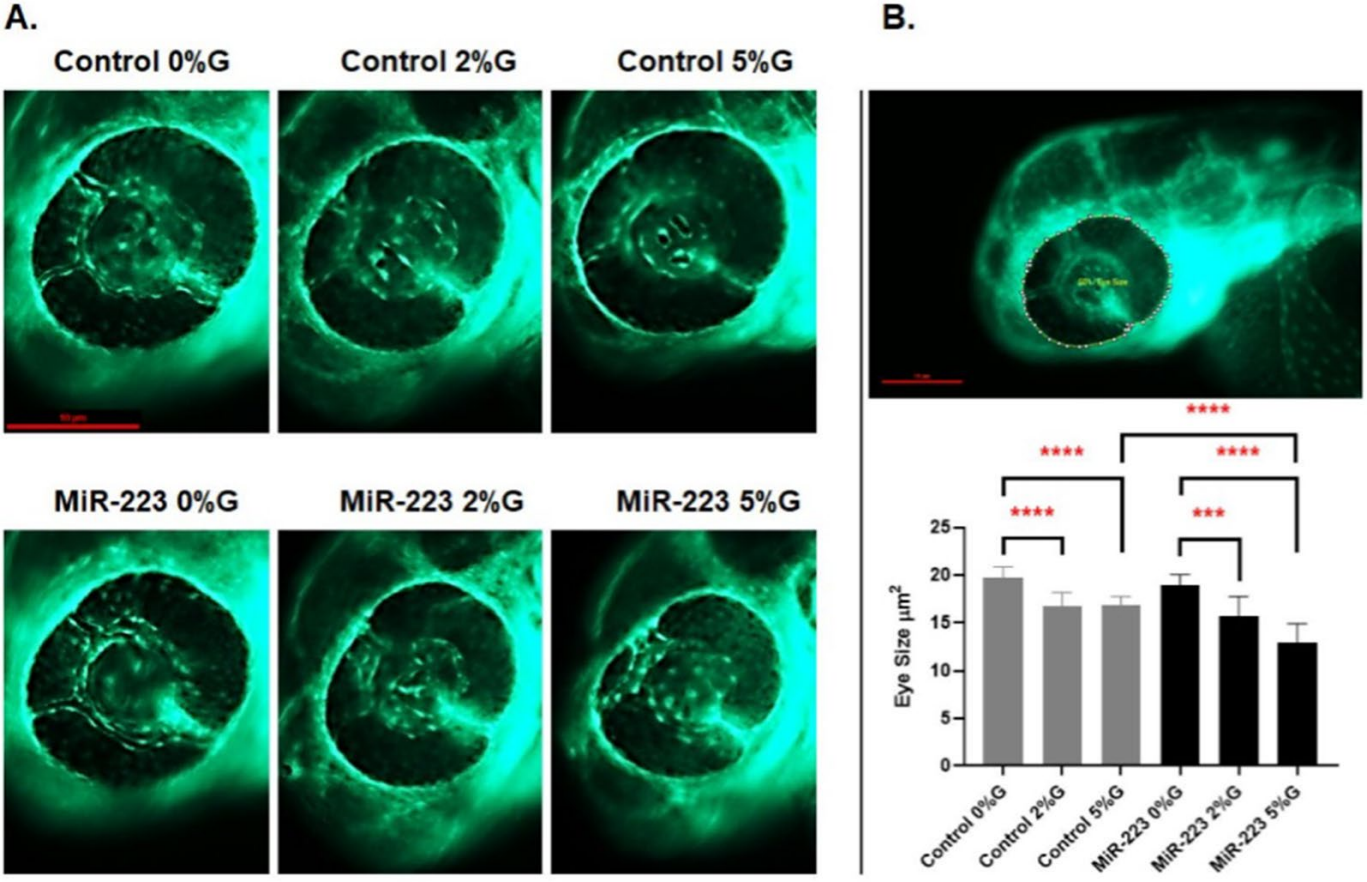
miR-223-3p association with zebrafish eye defects. **A** the different groups were imaged at ×150 magnification, and the eye images were traced using danioscope software (Noldus Technologies, Netherlands) for eye size measurement. Images were obtained using Lumar 12 stereomicroscope (Zeiss Microscopy) and a Nikon camera at ×100 magnification; scale bar, 10 mm. **B** incubation of the different groups in glucose affected the development of the eyes, as demonstrated by aberrant vasculature nourishment that led to a significant reduction in eye size. The different groups incubated in 2% and 5% glucose showed significantly reduced eye size on day 3. The miR-223-3p mimic had severely impaired eye morphology compared to the controls in the 5% glucose condition. Total number of larvae measured was n = 10 per group. Statistical analysis was conducted using ANOVA Graph Pad version 9.0
- Histological changes in the eye at the cellular level
  Histological sectioning of the zebrafish eye displayed normal cellular morphology: the ganglion cell layer, inner nuclear layer, and outer nuclear layer in controls. Injection of miR-223-3p mimic and induction of hyperglycemia both resulted in altered eye tissue morphology. The control and miR-223-3p groups at 2 and 5%, and 0%, 2%, and 5%, respectively showed distinct abnormal changes in the eye morphology, layer thickness, and cellular components in the retina (Fig. 6).

- Differences in vasculature branching in the eye
  The association between miR-223-3p and DR was evident upon injecting miR-223-3p mimic in zebrafish, compared with control groups at 0%, 2%, and 5% glucose. MiR-223-3p had a severe negative effect on vascular branching, the most prominent at 5%. The eye vasculature was scored as severe, mild, and normal based on blood vessel sprouting. The blood vessels in miR-223-3p displayed abnormal sprouting (a severe eye disorder) on day 3 of development for 0%, 2%, and 5% glucose. The most severe sprouting defects accounted to 10% abnormality in the control group at 5% glucose and 10%, 50%, and 60% abnormality in the miR-223-3p mimic group at 0%, 2%, and 5% glucose, respectively (p = 0.02, p < 0.0001, and p < 0.05, respectively) (Fig. 4).
  The overexpression of miR-223-3p affected the eye vasculature sprouting through development. Remarkably, the miR-223-3p high levels in association with hyperglycemia conditions (2% and 5% Glucose), further worsen the eye vasculature sprouting to result in mild and severe effect (Fig. 4B, C). We observed a sharp rise in the number of larvae with severe developmental defects at higher doses of miR-223-3p titration (Fig. 4E), further supporting the role of miR-223-3p in inducing developmental defects and changes in eye morphology.
- Eye size
  Zebrafish eye diameters were measured to observe the impact of altered vessel sprouting on eye size. A significant reduction was observed in the eye size (µm^2^) and hence retinal development in the groups exposed to varying glucose conditions (induced hyperglycemia) compared to controls. The eye size of 3-day-old zebrafish in the control groups at 2% and 5% glucose conditions were 16.76 µm^2^ and 16.83 µm^2^, respectively, compared to 19.7 µm^2^ in 0% glucose, (p = 0.002 and p = 0.0002, respectively). A similar effect was observed in the miR-223-3p mimic injected group after hyperglycemia was induced. The eye sizes of 3-day-old zebrafish in the miR-223-3p mimic groups in the 2% and 5% glucose conditions were 15.7 µm^2^ and 12.97 µm^2^, respectively, while that of the miR-223-3p mimic at 0% glucose was 18.93 µm^2^, (p = 0.01 and p = 0.0001, respectively). Interestingly, elevated miR-223-3p mimic levels and hyperglycemic conditions (5% glucose) resulted in more significant impairment in eye growth compared to the control group at 5% glucose. In the miR-223-3p mimic group at 5% glucose, eye size was significantly reduced to 12.98 µm^2^ compared to the control group at 5% glucose (16.83 µm^2^, p < 0.001) (Fig. 5).

**Fig. 6:**
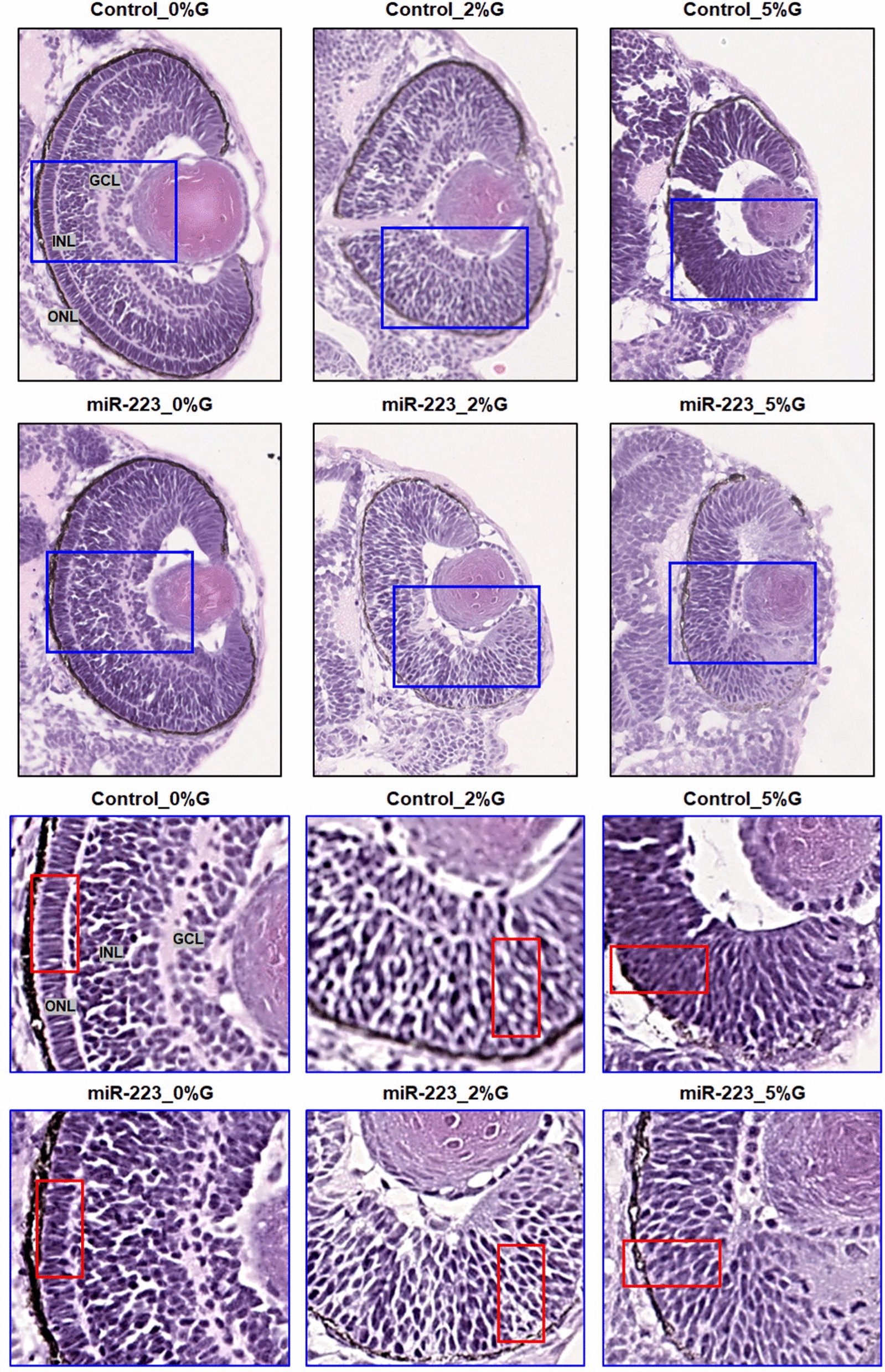
miR-223-3p association with abnormalities in the zebrafish eye. Histological examination was performed on coronal sections of whole zebrafish larvae that were stained with hematoxylin and eosin. Both miR-223-3p mimic and hyperglycemia conditions displayed abnormal eye morphology and eye cellular components, including the ganglion cell layer (GGL), inner nuclear layer (INL), and outer nuclear layer (ONL). The MiR-223-3p overexpression resulted in sparse cellular arrangements within the eye compared to matched glucose condition. All other groups demonstrated severe abnormalities in the cellular components of the eye. Images were obtained using a Philips slide scanner at ×40 magnification

#### RT-qPCR

**Fig. 7:**
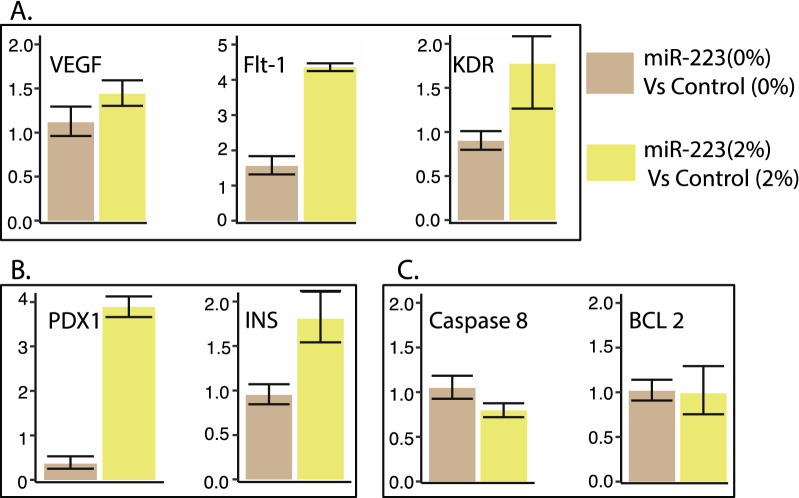
miR-223-3p mimic effects in zebrafish model. **A** Effects of miR-223-3p on mRNA expression of three angiogenic markers (VEGFA, FLT-1, KDR) in zebrafish embryos. The expression levels were normalized to EF1A with bars representing average relative expression from replicates and whiskers indicating standard errors of mean. The overexpression of miR-223-3p mimic (miR-223-3p) at 0% and 2% glucose conditions compared to the control group (Control). The increased levels of miR-223-3p resulted in a fold-change increase in the three angiogenic markers compared to matching control groups. **B** Effects of miR-223-3p on mRNA expression of two pancreatic markers (PDX1, required for the pancreas and beta-cell development) and (INS, regulator of blood glucose levels by insulin-secreting beta-cells) in zebrafish embryos. For both, the expression levels were normalized to EF1A. In the presence of 2% glucose, adding miR-223-3p resulted in a fold-change increase in the two pancreatic markers compared to the matching control group. **C** Effects of miR-223-3p on mRNA expression of two apoptosis markers (CASP 8 and BCL 2) in zebrafish embryos. For both markers, the expression levels were normalized to EF1A. The increased levels of miR-223-3p in the absence (0%) and the presence of 2% glucose showed comparable expression of the two markers compared to the matched control groups

**Fig. 8:**
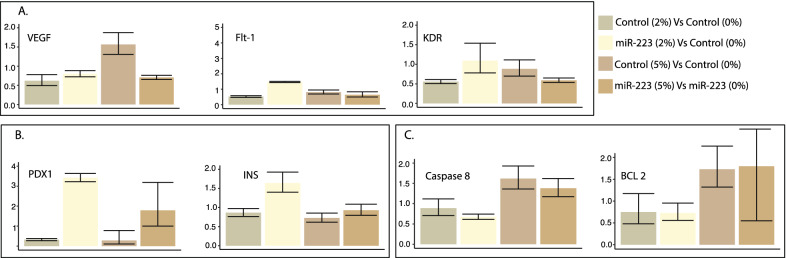
Hyperglycemic effects in zebrafish models. **A** The hyperglycemia conditions (2% and 5% glucose) altered angiogenic marker expression levels. Increased levels of miR-223-3p were associated with increased expression of VEGF receptors (FTL-1 and KDR) at 2% glucose condition compared to the control group. The Control group at 5% glucose condition showed increased VEGF expression levels. **B** The hyperglycemia conditions were associated with the upregulation of pancreatic markers within the mimic miR-223-3p group. **C** The control group showed increased expression levels of apoptotic markers at 5% glucose. Expression was analyzed as described in Fig. 7
- Increased miR-223-3p increases pancreatic marker ratios
  The expression of pancreatic markers PDX1 and INS increased by 3.88- and 1.81-folds, respectively, in the case of an increased miR-223-3p group compared to controls under similar conditions of 2% glucose (Fig. 7B). Similar increases in *PDX1* and *INS* expression levels (3.42- and 1.64-fold, respectively) were seen with increased miR-223-3p at the 2% and 5% glucose conditions (Fig. 8B).
- MiR-223-3p did not affect apoptotic markers
  Acridine orange staining to detect apoptotic cells in zebrafish embryos remained inconclusive. To overcome this, we performed qPCR for selected apoptosis markers *CASP8* (apoptosis and cell-survival mediator) and *BCL-2* (controls apoptotic pathways). The increased miR-223-3p levels did not affect the expression of the apoptotic markers (Fig. 7C). However, under 5% glucose, *CASP8* and *BCL-2* showed increased expression in control (1.62- and 1.38-fold, respectively) and miR-223-3p (1.73- and 1.8-fold, respectively) (Fig. 8C).

- MiR-223-3p detection
  The analysis showed a > eightfold increase in miR-223-3p levels in the miR-223-3p -injected group to the control group. Injecting miR-223-3p increased angiogenesis marker ratios. Elevated miR-223-3p showed increased expression of angiogenic markers, *VEGF*, *FLT-1* and *KDR* compared to controls at 2% glucose, confirming its role in zebrafish retinopathy in the developmental stage. The *VEGF* and *FLT-1* expression was 1.44 and 4.36-fold higher, in a miR-223-3p group compared to controls at 2% glucose concentration (Fig. 7A). Our results show a considerable association between increased miR-223-3p and glucose, confirmed by the overexpression of *VEGF* and its receptors, suggesting the interplay of miR-223-3p and glucose levels in diabetes and associated retinopathy (Figs. 7A, 8A).

## Discussion

miRNAs play crucial roles in multiple biological processes, including cell proliferation, differentiation, development, and apoptosis [46, 47]. Despite the implication of dysregulated miRNAs in many diseases, their role in diabetes and related complications is unclear. Although many miRNAs play vital roles in tissues with diabetic complications, their involvement in the damage is yet to be established. miR-223-3p, an Insulin-like growth factor 1 regulator, is crucial in maintaining functional β-cell mass via Foxo1 and Sox6 signaling cascades [30]. Ablation of miR-223-3p causes increased expression and nuclear localization of Foxo1, suppressing Pdx1 and liver glucose transporter (Glut2, the major glucose transporter in pancreatic β-cells and hepatocytes [48] while increasing of cyclin-dependent kinase inhibitor (p27 protein) levels [49]. Studies in DR retinal endothelial cells from a DR rat model revealed an association between miR-223-3p expression, miR-223-3p -modulated cell proliferation, and high-glucose-induced cell proliferation, in which miR-223-3p negatively regulates eukaryotic translation initiation factor 4E family member 3 (*EIF4E3*) and Insulin-Like Growth Factor 1 Receptor (*IGF1R*) [31]. Further, functional analysis in 3T3-L1 cells (murine-derived pre-adipocytes) revealed impaired glucose and lipid metabolism upon increased intracellular expression of miR-223-3p [50]. In this study, miR-223-3p was upregulated in our cohort of Qataris with T2D.

We used open array miRNA profiling to establish the first miRNA signature biomarkers in T2D and DR in a Qatari population. Our study highlights the dysregulation of multiple miRNAs in type 2 diabetes, with significantly elevated expression of miR-223-3p (also associated with HbA1c and glucose) in DR patients suggesting its role as a biomarker and therapeutic target in DR/at-risk type 2 diabetic patients. It is interesting to note that whereas miR-223-3p levels positively correlated with HbA1c (1.64e−05) and glucose (9.88e−04), we found a statistically insignificant albeit a negative correlation with C-peptide and Insulin, representing a novel aspect of our study that is worth further investigation. Others have explored the association between C-peptide and circulating miR-223-3p alongside other miRNAs in patients with T1D versus healthy controls. The expression level of miR-223-3p was substantially increased in diabetic patients versus controls in correlation with C-peptide levels [51]. Our results support that miRNA assessment may be a useful addition to the routine diagnosis particularly given that genetics, environmental and lifestyle measures differ by ethnicity.

In the obesity context, circulating miR-223-3p was reported to be associated with diabetes independent of obesity and BMI status in affected individuals [52]. In addition, miR-223-3p displayed significant correlations with glycemic control and β cell function measures in subjects with T2D including HbA1c, fasting glucose, glucose AUC, 2 h insulin, HOMA B and C-peptide, along with waist circumference, fat mass, fat percentage, lean mass, total weight in lean and obese, diabetic, and pre-diabetic individuals [53]. In combination with other miRNAs, miR-223-3p presented a higher predictive value in the T2D diagnosis than the standard glycemic control clinical measures individually (HbA1c and fasting glucose, and 2 h glucose of oral glucose tolerance test (OGTT) [54]. These data suggest that alteration in circulating miR-223-3p in correlation with other miRNAs and mediators may have clinical value as a screening biomarker to identify subjects with prediabetes at increased risk of developing diabetes [55].

The role of miR-223-3p as a potential biomarker in retinal diseases and its effects on T2D and/or DR progression has been investigated. Studies have shown that miR-223-3p is necessary to preserve normal retinal function and regulate inflammation. Determination of miR-223-3p targets and their vital biological interactions relevant to retinal diseases is important. In a mouse model, in photo-oxidative damage-induced degeneration condition, miR-223-3p was elevated in the retina, circulating serum, and retinal extracellular vesicles demonstrating that miR-223-3p is required for maintaining normal retinal function. Retinal function in miR-223-3p^–/–^ mice were severely affected, indicating the regulatory effect of retinal microenvironment response and both delivery of miR-223-3p mimics enhanced retinal function in mice experiencing retinal deterioration and suggesting a dual role of miR-223-3p in retinal function and development [56]. Further, in a rabbit model, miR-223-3p mimics stimulated the expression of apoptosis and inflammation factors and inhibited cell proliferation by targeting HSP-70 due to the loss of retinal ganglion cells in Glaucoma, which might represent a new treatment strategy for the disease [57].

The role of miR-223-3p in diabetes complications have been widely discussed. In this context, miR-223-3p may play a role in oxidative stress regulation by interacting with cellular responses against microenvironment stresses. Nrf2 (NF-E2-related factor 2) is a regulator of cellular stress through stimulating antioxidant enzymes and detoxification factors. The activity of Nrf2 is regulated by Keap1 (Kelch-like ECH-associated protein 1), an adaptor of Cullin 3-based E3 ubiquitin ligase [58]. Overexpression of miR-223-3p activates the keap1-Nrf2 system via the involvement of ellagic acid (need a reference here). MiR-223-3p, a negative regulator of keap1 represents an attractive therapeutic focus in hepatic injury in T2D.

MiR-223-3p has been characterized as the dormant therapeutic target for diabetic cardiomyopathy. MiR-223-2p was over-expressed in the hyperglycemia-induced cardiomyocyte injury cellular model; in addition, inhibiting miR-223-3p expression led to further activation of myocardial fibrosis and specific apoptotic pathways, including the attenuation of NLRP3 inflammasome in diabetic cardiomyopathy [59].

To understand the role of miR-223-3p in the development of DR in T2D patients, we established a zebrafish hyperglycemia model to investigate its effect in the presence of high glucose levels on retinal development. The overexpression of miR-223-3p resulted in a remarkable increase in total glucose levels in zebrafish incubated under high glucose conditions for 3 days, establishing a relationship between miR-223-3p and glucose homeostasis. It was reported earlier that miR-223-3p overexpression in human cardiomyocytes and adipocytes enhances the overall glucose uptake by regulating genes and proteins involved in glucose homeostasis [60]. Our evaluation of pancreatic function in the developing zebrafish showed upregulated expression of both pancreatic markers, *PDX1* and *INS*, by 3.8- and 1.8-fold, respectively, in the miR-223-3p mimic group. Pdx1 is a transcription factor that plays a central role in pancreatic β-cell function and survival [61]. Abnormal expression of Pdx1 leads to β-cell dysfunction and possible abnormal vessel morphology and sprouting [62]. Also, upregulation of miR-223-3p increases insulin resistance through upregulation of Glucose transporter type 4 (GLUT4) protein, the major regulator of insulin-mediated glucose translocation into the adipocytes [63]. Also, aberrant retinal vascular formation is observed in a *pdx1*−/− mutant zebrafish [64]. Here, we report that upregulation of *PDX1* is related to miR-223-3p mimic injection under hyperglycemia conditions. Our results are similar to earlier reports showing that under high-glucose concentrations, increased PDX1 enhanced insulin gene expression [65]. Collectively, in our model, miR-223-3p alters glucose hemostasis and pancreatic function and is associated with increased total glucose, pdx1, and insulin expression in developing zebrafish.

Our model demonstrates the effect of miR-223-3p overexpression in the pathogenesis of vascular branching by severely affecting eye nourishment. MiR-223-3p increased levels led to abnormal vasculature in 80% of the zebrafish larvae, which was confirmed at the molecular level. The expressions of selected genes were elevated in the VEGF pathway upon injecting miR-223-3p. It is known that VEGF binds and activates the receptors VEGFR-1 (*FLT-1*) and VEGFR-2 (*KDR*) to initiate the VEGF pathway [66]. The expression levels of the three genes examined, *VEGF*, *FLT-1*, and *KDR* were upregulated in the miR-223-3p mimic group at both 0% and 2% glucose conditions. However, the expression levels at 5% glucose were inconclusive, suggesting that the high glucose dose may result in a global effect at the transcriptional level. Our results indicate that miR-223-3p influences VEGF signaling pathways possibly resulting in angiogenic abnormalities in the zebrafish developing eyes. The changes in the developing eye vasculature resulted in a significant reduction in the eye area size in both the zebrafish miR-223-3p mimic group and the hyperglycemia-induced group.

Our results are in accordance with the previous reports wherein *FLT-1* staining was observed in both non-diabetic and diabetic vascular and extravascular retinal tissue; increased immunostaining was observed in preretinal and intraretinal vessels of diabetic tissue compared with non-diabetic tissue [67]. Additionally, our model demonstrated that miR-223-3p overexpression resulted in abnormal eye tissue structures, including altered thickness and sparse cellular arrangements of the inner nuclear layer (INL) layer; likewise, induction of hyperglycemia had similar results. The alteration of INL thickness is a characteristic of retinal development under high glucose [66]. Thickening of the INL layer may induce progressive neural loss, subsequent loss in vision, and increased apoptosis [66, 68]. Our results confirmed the effect of hyperglycemia in different tissues, indicating upregulation of apoptotic markers (BCL and caspase 8). Interestingly, there was no effect of miR-223-3p on the apoptotic markers studied, which is otherwise reported to be vital in regulating apoptosis in multiple tissues [31, 69]. We hypothesize that miR-223-3p has no effect on apoptosis at early retinal development and that it affects the same in later stages. This is in accordance with reports that visual loss associated with DR is due to apoptosis-induced loss in photoreceptors [70, 71]. This dual effect of hyperglycemia and miR-223-3p possibly contributes to the aberrant eye vasculature formation that may lead to a subsequent increased apoptosis and retinal cell death.

Collectively, our functional validation established the in vivo association between increased miR-223-3p, glucose hemostasis and ocular development.

Our model established that high miR-223-3p levels triggered aberrant eye development events, including vascular sprouting and changes in retinal cellular components that might lead to DR in diabetic individuals, suggesting the role of miRNAs in diabetes-associated complications. Our established zebrafish model demonstrated that elevated miR-223-3p levels resulted in higher glucose, altered insulin expression, and altered pdx1 expression. Together, our model established the novel mechanistic of the combined effect of elevated miR-223-3p and hyperglycemia condition that contributed to the abnormal ocular vasculature that subsequently may lead to enhanced apoptosis and retinal cell death in DR.

## Conclusions

So far, there are few clinical studies and several in vitro animal studies aimed at understanding the dysregulated miRNAs in DR. Most studies carried out are candidate miRNAs studies that use retinal tissues from various animal models or endothelial cells exposed to high glucose conditions in vitro [72–82]. Very few studies have used clinical samples [83–87]. Although one study demonstrated the role of two miRNAs as predictors of diabetic retinopathy [86]—this study was carried out in a selected smaller subset of clinical trial/study samples and focused on miRNAs that the team had previously identified to be important in cardiovascular diseases and T2D (but not specific to DR). In this study, we used ultra-detailed phenotypic and clinical data to identify miRNA signatures that are correlated with glycemic control and β cell function measures, in a T2D cohort that reflects the features of the entire population. In addition, an established molecular miRNA signature of vascular damage identified by the study team in the blood of individuals with proliferative retinopathy is being characterized in this study. There is a need to follow up by examining the potential of these and similar molecular markers of DR in a larger clinical cohort, across different ethnic groups, and in different types of diabetes covering a wide age range/stages of DR. The effects of clinical, pharmacologic, and diabetes-related interventions on miRNA profiles and their relationships to clinical endpoints are of great interest. Since miRNAs themselves may be therapeutic targets or even (as anti-miRs) a therapeutic agent [81], such studies would stimulate more investigation to understand the therapeutic potential of miRNAs for treating retinopathy in individuals with diabetes.

## Supplementary Information

**Additional file 1: Figure S1.** Conservation of miRNA 223. **Figure S2.** Zebrafish miR-223 functional model survival rate. **Figure S3.** Zebrafish miR-223 mimic expression. **Table S1.** Demographic data and clinical characteristics of study subjects. **Table S2.** Primers used for gene expression evaluation in zebrafish.

## Data Availability

Materials described in the manuscript, including all relevant raw data, will be freely available to any scientist wishing to use them for non-commercial purposes without breaching participant confidentiality. Additional file data contains input files and the R script to analyze gene expression data and visualization of results.
