# Supplementary material for: The link between glycemic control measures and eye microvascular complications in a clinical cohort of type 2 diabetes with microRNA-223-3p signature
Source: J Transl Med. 2023 Mar 3;21:171. doi: 10.1186/s12967-023-03893-2 (PMC9985290; doi:10.1186/s12967-023-03893-2)
Supplement: Supplementary file 1 — Additional file 1: Figure S1. Conservation of miRNA 223. Figure S2. Zebrafish miR-223 functional model survival rate. Figure S3. Zebrafish miR-223 mimic expression. Table S1. Demographic data and clinical characteristics of study subjects. Table S2. Primers used for gene expression evaluation in zebrafish. [file 12967_2023_3893_MOESM1_ESM.docx]

**Additional file 1**

**The Link Between Glycemic Control Measures and Eye Microvascular Complications in a Clinical Cohort of Type 2 Diabetes with MicroRNA-223-3p Signature**

**Figure S1. Conservation of miRNA 223**

Conservation of human miR-223-3p and zebrafish (*Danio rerio*) miR-223

**
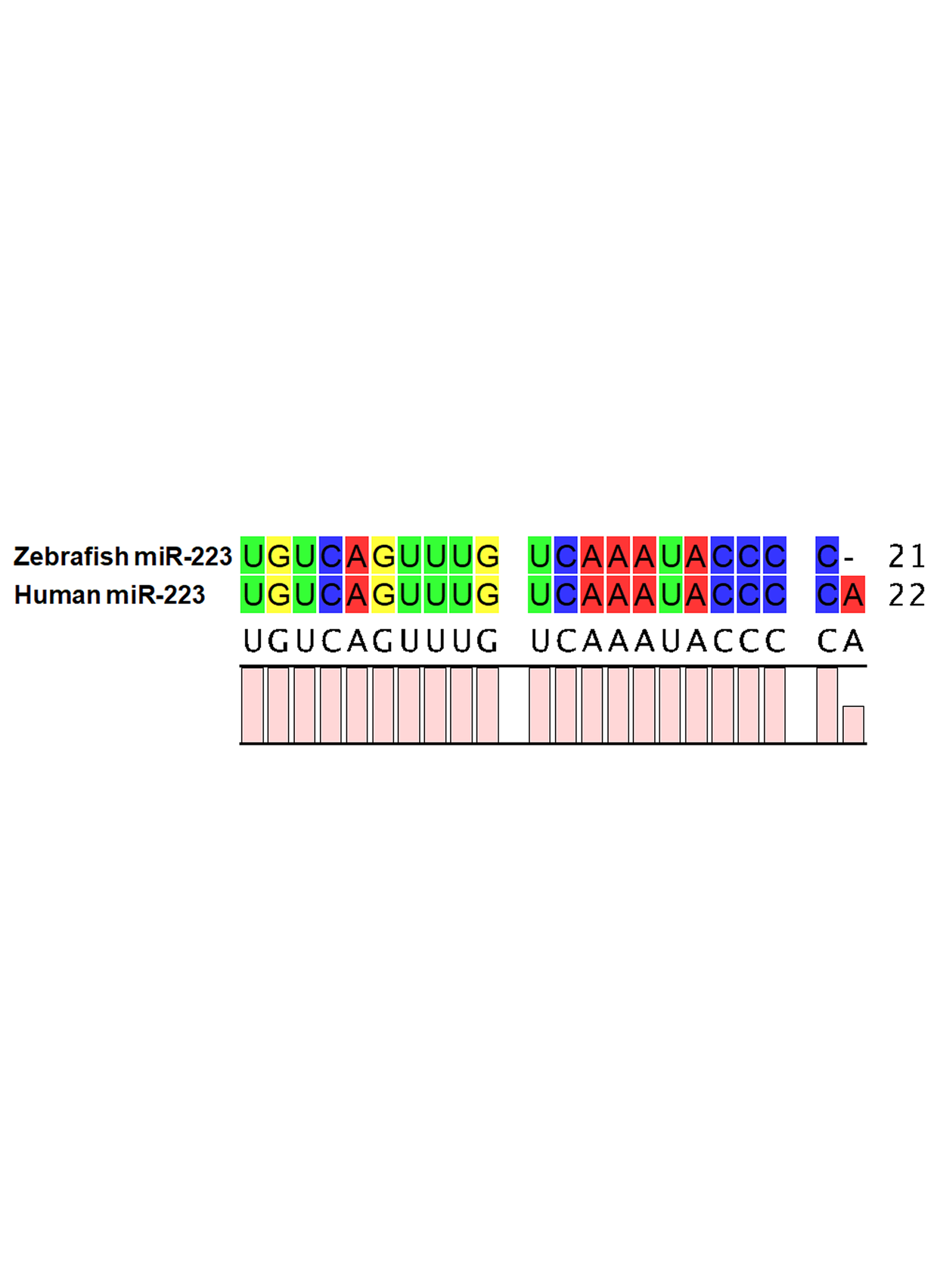
**

**
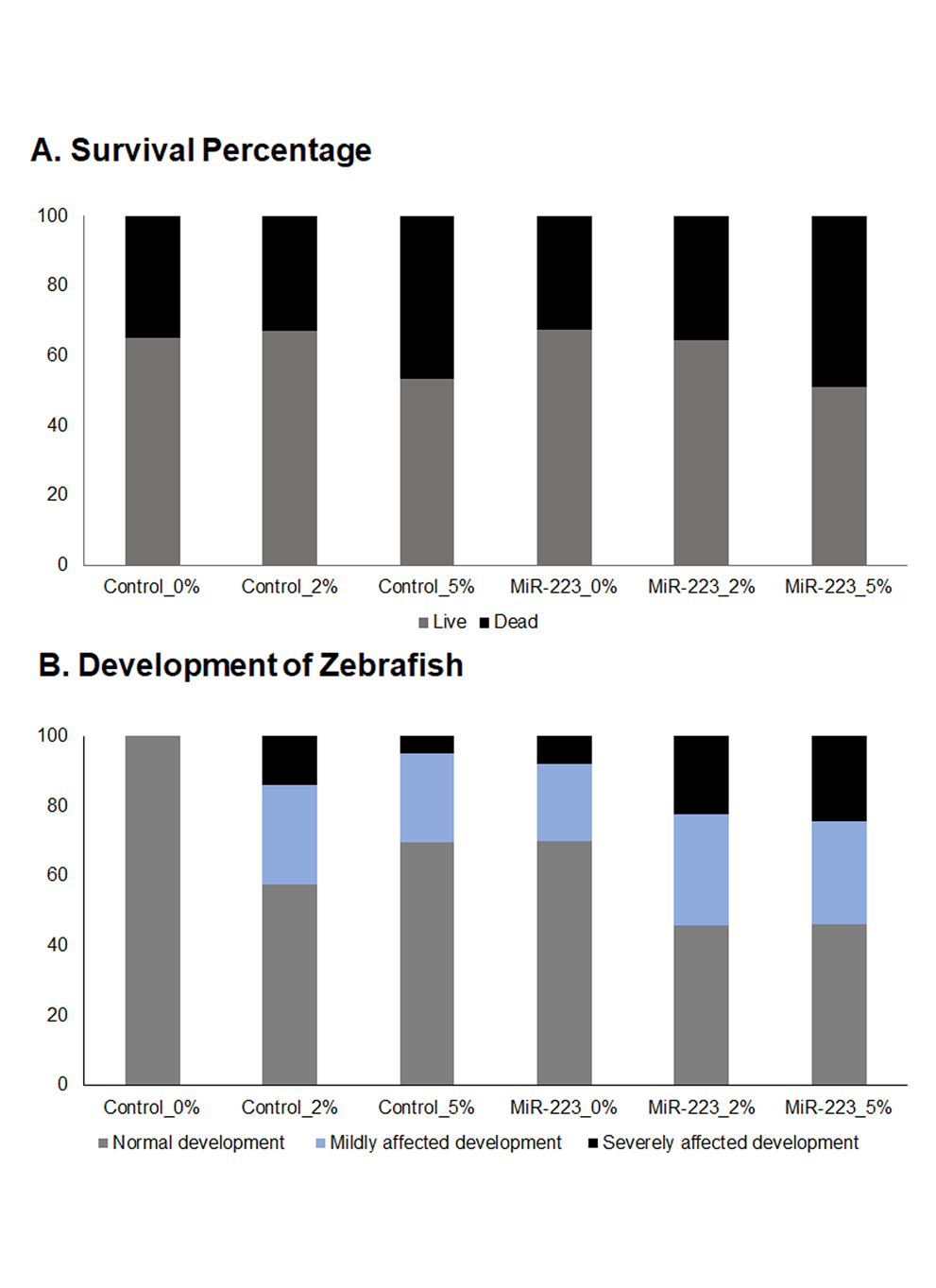
**

**Figure S2. Zebrafish miR-223 functional model survival rate**

**A.** Injection of miR-223 mimic into one-cell stage zebrafish embryos had no effect on zebrafish survival rate compared to control fish in the corresponding glucose conditions. The induction of hyperglycemia had a negative effect on zebrafish survival (Figure 1). Statistical analysis using chi-square was performed to compare zebrafish survival rate among groups (Control 0% Glucose, C_0%; Control 2% Glucose, C_2%; Control 5% Glucose, C_5%; miR-223 mimic injected in 0% Glucose, M_0%; mimic injected in 2% Glucose, M_2%; mimic injected in 5% Glucose, M_5%).
**B.** Injection of miR-223 mimic (M_0%) into one-cell stage zebrafish embryos had a significant effect on zebrafish development compared to the control group (C_0%). The classification of zebrafish development was as follows: G1, severely affected development; G2, mildly affected development; G3, normal development. Statistical analysis was conducted using chi-square.

| Condition | miR223 relative expression (ref Control 0%G) |
| --- | --- |
| Control 0% G | 1.000 |
| Control 2% G | 0.7149 |
| Control 5% G | 0.076 |

**Figure S3. Zebrafish miR-223 mimic expression**

The extracted total RNA from the control groups was used to perform quantitative RT-PCR using TaqMan microRNA assay stem-loop primers for mature miR-223 detection. Under glucose conditions, miR-223 expression levels were reduced compared to the control group at 0% glucose.

| **Table S1:** Demographic data and clinical characteristics of study subjects. Data in this table represent average values along with the standard error of the mean. HbA1c, hemoglobin A1c; C-peptide, proinsulin connecting peptide; INS, insulin; LDL, low-density lipoprotein; HDL, high-density lipoprotein; CHO, total cholesterol; BMI, body mass index. |
| --- |

|  | AGE | HbA1C  (%) | C-peptide  (ng/mL) | INS  (mIU/L) | LDL  (mmol/L) | HDL  (mmol/L) | CHO  (mmol/L) | Triglycerides  (mmol/L) | BMI |
| --- | --- | --- | --- | --- | --- | --- | --- | --- | --- |
| Diabetic males (n=223) | 49±0.73 | 7.63±0.12 | 3.53±0.17 | 23.7±2.08 | 2.9±0.06 | 1.11±0.02 | 4.86±0.07 | 1.95±0.08 | 29.8±0.39 |
| Diabetic females (n=248) | 51±0.66 | 7.39±0.12 | 3.18±0.11 | 24.8±3.04 | 2.83±0.06 | 1.36±0.02 | 4.97±0.07 | 1.68±0.05 | 32.5±0.43 |
| Control males (n=284) | 36±0.60 | 5.31±0.02 | 3.02±0.13 | 18.5±1.42 | 3.11±0.05 | 1.2±0.02 | 4.96±0.06 | 1.49±0.08 | 28.3±0.35 |
| Control females (n=207) | 36±0.84 | 5.33±0.03 | 2.43±0.1 | 12.1±0.72 | 2.82±0.05 | 1.53±0.02 | 4.87±0.06 | 1.13±0.04 | 28.9±0.49 |

| **Table S2**. Primers used for gene expression evaluation in zebrafish |
| --- |

| Target | Forward primer | Reverse Primer |
| --- | --- | --- |
| *vegf* | TGCTCCTGCAAATTCACACAA | ATCTTGGCTTTTCACATCTGCAA |
| *caspase 8* | GATCGAGAGGTTCAGGAATCAGA | CATTGTTTCAGATACAGGGTTGTTG |
| *bcl* | CGGCCAACAGCTGAGAAGA | GCTGGGCGATTGACTCATCT |
| *flt-1* | ATGGGAACAGCAGCACTCTT | TTGAAGACGGAGGGACAATC |
| *kdr* | TGTGGTCAGCTATGCTGGAG | AGCCTCTCATGCTGTGGACT |
| *pdx1* | TTTCCCCGGTCTATGGCAAT | TGGCCAAAGTACGA GTTACCT |
| *ins* | AAGCACTAACCCAGGCACAC | GAAGGGGCTCAACGTCTCTC |
| *ef1a* | GAGGAAATCACCAAGGAAGTCAG | TTGAACCAGCCCATGTTTGAG |
